# Alterations of RNA splicing patterns in esophagus squamous cell carcinoma

**DOI:** 10.1186/s13578-021-00546-z

**Published:** 2021-02-09

**Authors:** Jiyu Ding, Chunquan Li, Yinwei Cheng, Zepeng Du, Qiuyu Wang, Zhidong Tang, Chao Song, Qiaoxi Xia, Wenjing Bai, Ling Lin, Wei Liu, Liyan Xu, Enmin Li, Bingli Wu

**Affiliations:** 1 Key Laboratory of Molecular Biology for High Cancer Incidence Coastal Chaoshan Area, Shantou University Medical College, Shantou, 515041 China; 2 Department of Biochemistry and Molecular Biology, Shantou University Medical College, Shantou, 515041 China; 3 School of Medical Informatics, Harbin Medical University, Daqing Campus, Daqing, 163319 China; 4 Department of Pathology, Shantou Central Hospital, Affiliated Shantou Hospital of Sun Yat-sen University, Shantou, 515041 China; 5 Institute of Oncologic Pathology, Shantou University Medical College, Shantou, 515041 China

**Keywords:** Alternative splicing, MISO, Esophagus squamous cell carcinoma

## Abstract

Alternative splicing (AS) is an important biological process for regulating the expression of various isoforms from a single gene and thus to promote proteome diversity. In this study, RNA-seq data from 15 pairs of matched esophageal squamous cell carcinoma (ESCC) and normal tissue samples as well as two cell lines were analyzed. AS events with significant differences were identified between ESCC and matched normal tissues, which were re-annotated to find protein coding genes or non-coding RNAs. A total of 45,439 AS events were found. Of these, 6019 (13.25%) significant differentially AS events were identified. Exon skipping (SE) events occupied the largest proportion of abnormal splicing events. Fifteen differential splicing events with the same trends of ΔΨ values in ESCC tissues, as well in the two cell lines were found. Four pathways and 20 biological processes related to pro-metastasis cell junction and migration were significantly enriched for the differentially spliced genes. The upregulated splicing factor SF3B4, which regulates 92 gene splicing events, could be a potential prognostic factor of ESCC. Differentially spliced genes, including HNRNPC, VCL, ZNF207, KIAA1217, TPM1 and CALD1 are shown with a sashimi plot. These results suggest that cell junction- and migration-related biological processes are influenced by AS abnormalities, and aberrant splicing events can be affected by splicing factor expression changes. The involved splicing factor SF3B4 was found to be a survival-related gene in ESCC and is presumed to regulate AS in multiple cancers. In summary, we identified significant differentially expressed AS events which may be related to the development of ESCC.

## Introduction

Esophageal cancer is the sixth leading cause of cancer fatalities in the world [1], with a five-year survival of less than 20% [2]. Esophageal cancer is mainly classified into two types that differ in epidemiology and pathology: esophageal squamous cell carcinoma (ESCC) and esophageal adenocarcinoma (EAC). About 90% of esophageal cancer cases worldwide are ESCC [1]. Studies committed to the molecular mechanism of ESCC development need to be carried out further to help improve the diagnosis, treatment and prognosis of this detrimental disease.

Pre-mRNA splicing is a biological process, carried out by a spliceosome consisting of five snRNP complexes and more than 200 auxiliary proteins [4], that removes intron lariats and joins exons together to produce mature mRNAs [3]. In the process of alternative splicing, alternative splice site selection within pre-mRNAs results in the production of various mature mRNAs from a single gene [5]. It has been reported that alternative splicing plays important roles in normal organ development and cell differentiation [6]. However, splicing pattern abnormalities caused by mutations of cis-acting sequences and spliceosomal proteins can also trigger human diseases, including cancer [7, 8]. Altered splicing patterns influence multiple aspects of cancer development including cell proliferation, programmed cell death, metabolism, angiogenesis, and tumor metastasis [5, 9]. Genome-wide studies indicate that tumor development often involves large-scale variations in alternative splicing [10].

Recent developments in high-throughput sequencing technologies and computational tools have revolutionized our capacity to investigate AS at the genome-wide level. In 2018, changes in exon usage were found across the full TCGA cohort, where tumor samples had an increase in abnormal splicing events as compared to normal samples for the majority of the investigated cancers [11]. Using PSI (percent spliced in) and DEXSeq methods, 28 pairs of colorectal cancer and normal tissues were analyzed, where 9 significant AS genes were found with two splicing events associated with patient overall survival [12]. Applying public data from TCGA, more than 200 DEGs (Differentially Expressed Genes) with AS events were identified and a risk score based on 12 genes associated with overall survival was established in colon adenocarcinoma[13]. In kidney renal clear cell carcinoma, 13 survival-specific genes were significantly correlated with AS events from SpliceSeq database [14].

The MISO model was originally developed to assess the expression of alternatively spliced exons and transcripts [15]. MISO uses confidence intervals to assess exon and transcript abundance, but also detects differentially-spliced exons or transcripts. This is accomplished by calculating the posterior distribution of unobserved random variables from the RNA-Seq data [15]. The combination of RNA-seq data and computational tools enables AS analyses at a genome-wide level, which provides insight into AS mechanisms and potential clinical application. In this study, MISO was utilized to characterize the splicing patterns and try to identify the regulatory roles of splicing factors on splicing abnormalities in ESCC.

## Materials and methods

### Source of sequencing data and routine analysis

RNA-seq data of 15 pairs of ESCC tissues was generated from our laboratory, which has been published in the previous study [16]. FastQC was used to measure the quality of the sequences [17]. The paired-end sequences were then aligned against the UCSC (http://genome.ucsc.edu/) human genome (hg19) [18] using TopHat2 v2.0.13 [19] and Bowtie2 [20] using default parameters. Alignments were further analyzed with Cufflinks [21] to calculate the abundance (FPKM) of each gene and isoform using the hg19 gene annotations, and with DESeq [22] to identify differentially-expressed splicing factors. To validate the differentially-expressed splicing factors, two microarray datasets, named GSE53624 [23] and GSE23400 [24], were utilized to perform differential expression analysis with the siggenes package in R2.15.3. The RNA-seq data from a pair cell lines, an immortalized human esophageal epithelial cell line (SHEE) and its malignant transformed cell line (SHEEC) [16], was applied as the test cohort in this study.

### Identification of AS events in ESCC

The splicing patterns of ESCC were analyzed with MISO [15] using exon-centric annotations V.2.0, and the results were filtered with a cut-off of ΔΨ > 0.2, Bayes-factor > 5, and a sum of inclusion and exclusion reads number > 10. Sashimi plots were used to visualize AS events with significant differences which occurred in ≧ 3 pairs of ESCC tissues, and between the SHEE and SHEEC cell lines as well.

### Re‐annotation of differential AS events

AS events used in the previous analysis were annotated with human genome (hg19) alternative events v2.0, and their annotations were derived from Ensembl genes (Ensembl database), knownGenes (UCSC database) and RefSeq genes (NCBI database). In order to further verify the AS event annotations and find out their represented genes and transcripts, AS events with significant differences in at least 3 pairs of ESCC tissue samples were re-annotated. Homo_sapiens.GRCh37.75.gtf from the Ensembl database [25] and GRCh37_latest_genomic.gff from the RefSeq database [26] were chosen for re-annotation. Since the mRNA sequences and gene-related annotations of the UCSC database were derived from GenBank and RefSeq databases, those annotations were not adopted for re-annotation of AS events with several criterion described as follows: (i) if three exons in an SE (Skipped exon) event appear in the same transcript, the SE event is judged to be corrected, (ii) if two exons in an RI (Retained intron) event are present in the same transcript and there are no other exons between these two exons, then the RI event annotation is determined to be corrected, (iii) if adjacent exons, for example exons 1-2-4 and 1-3-4, occur in the same transcript in an MXE (Mutually exclusive exon) event, and exons 1–4 do not appear in any one transcript, the MXE event is judged as correct, (iv) if the variable exon (the longer or the shorter exon) and constitutive exons in an A3SS (Alternative 3’splice site) event occurs in the same transcript, and between these two exons there are no other exons, the A3SS event annotation is determined to be corrected, (v) if the variable exon and the constitutive exon in an A5SS (Alternative 5’splice site) appear in the same transcript, and between the two exons there are no other exons, the A5SS event annotation is judged to be correct. Since there are no chromosome numbers in the RefSeq exon annotations, the chromosomal locations of exons using RefSeq annotations should be checked later.

### Functional enrichment analysis

DAVID online tools [27] were utilized to perform functional enrichment analysis towards GO biological process and KEGG pathways. The significant cut-off was set to *P* < 0.05. Dot plots from R package named ggplot2 [28] were then applied to visualize the significantly enriched GO terms and KEGG pathways.

### **Differential AS events occurred both in ESCC tissues and SHEE/SHEEC cell lines**

After the results of differential AS events from the SHEE and SHEEC cell lines were filtered, the following information, including AS event names, Ψ values from the SHEE cell line, Ψ values from the SHEEC cell line and ΔΨ values from SHEE/SHEEC cells were selected. The re-annotation results of the AS events in ESCC tissues using gene annotations were overlapped with SHEE/SHEEC AS events, then the similar trend of ΔΨ values of each overlapped AS event in tissues and in cell lines was judged.

### Splicing regulatory network establishment

Gene expression of each sample was combined and merged with the splicing factor list, which is available from the SpliceAid2 database, which contains 2,220 target sites of 62 human splicing proteins and their expression data in 320 tissues [29]. Spearman correlation tests were performed between splicing factors and the splicing event/genes, by the calculation of the expression levels and the PSI values, accordingly. The significant pairs of splicing factors and splicing genes were chosen to establish a splicing regulatory network. Cytoscape software was used to visualize the splicing regulatory network, which is an open source platform for visualization of molecular interaction [30].

### Survival analysis

The expression and clinical data of 87 ESCC patients was obtained from the Xena browser database (https://xenabrowser.net/datapages/). The log-rank (Mantel-Cox) test was used to perform survival analysis and a Kaplan-Meier curve was plotted using GraphPad software.

### Statistical analysis

Statistical analyses were performed using SPSS 19.0 (IBM, Chicago, IL, USA) or R 3.15.3 (Auckland, New Zealand) for Windows. Comparisons of the relative expression of between paired tumor and non-tumor tissues, were performed using a paired *t*-test. Overall survival time was calculated by the Kaplan–Meier method and analyzed by the log-rank test. A two-tailed *P*-value < 0.05 was considered to be statistical significant.

## Results

### Overall mapping rate after genome alignments

Reads from the RNA-seq of each esophageal clinical case were mapped to human genome sequences. The results are shown in Table 1, where the total alignment number of the sequenced fragments ranged from 15 to 39 million, and the comparison rate ranged between 83 and 92%, suggesting the high reliability of these RNA-seq data.

**Table 1:** Summary of TopHat2 genomic alignments

Samples	Total reads		Mapped reads		Overall reads	
	**Left**	**Right**	**Left**	**Right**	Mapping_rate (%)	**Aligned_pairs**
B782N	51,906,891	51,906,891	45,391,832	42,285,862	84.50	39,419,636
B782T	20,187,921	20,187,921	18,022,068	16,864,424	86.40	15,851,609
B783N	22,772,025	22,772,025	20,876,811	20,292,461	90.40	19,220,668
B783T	22,026,613	22,026,613	20,199,656	19,665,894	90.50	18,669,500
B785N	23,881,681	23,881,681	21,879,991	20,946,206	89.70	19,806,614
B785T	27,545,958	27,545,958	25,310,820	24,617,731	90.60	23,314,980
B786N	19,526,756	19,526,756	18,043,943	17,643,909	91.40	16,752,687
B786T	20,424,033	20,424,033	18,841,925	18,319,670	91.00	17,453,969
B788N	22,166,170	22,166,170	20,319,140	19,346,438	89.50	18,276,699
B788T	19,329,401	19,329,401	17,949,865	17,468,161	91.60	16,651,551
B791N	25,487,339	25,487,339	22,803,788	22,091,751	88.10	20,629,163
B791T	19,730,469	19,730,469	17,587,161	16,688,030	86.90	15,713,877
B794N	21,773,037	21,773,037	20,089,962	18,134,197	87.80	17,197,455
B794T	27,630,784	27,630,784	24,796,070	22,364,260	85.30	20,946,236
B797N	24,393,756	24,393,756	22,517,747	21,882,580	91.00	20,713,525
B797T	20,082,035	20,082,035	18,745,753	18,269,290	92.20	17,451,339
B798N	32,153,939	32,153,939	29,786,198	26,930,352	88.20	25,572,896
B798T	32,468,547	32,468,547	30,246,172	27,414,170	88.80	26,154,479
B799N	25,194,910	25,194,910	23,448,172	21,151,887	88.50	20,095,525
B799T	20,897,751	20,897,751	19,481,307	17,526,890	88.50	16,701,290
B800N	26,328,835	26,328,835	24,474,467	22,048,959	88.40	20,927,261
B800T	24,186,510	24,186,510	22,481,444	20,362,666	88.60	19,317,914
B801N	22,799,054	22,799,054	21,390,618	19,363,520	89.40	18,518,368
B801T	26,946,581	26,946,581	25,259,555	22,713,737	89.00	21,717,932
B804N	27,121,722	27,121,722	25,391,238	22,801,422	88.80	21,774,130
B804T	24,985,226	24,985,226	23,383,899	21,149,641	89.10	20,212,413
C199N	29,776,640	29,776,640	26,261,213	25,254,804	86.50	23,366,546
C199T	35,651,673	35,651,673	30,502,787	29,013,594	83.50	26,708,316
C200N	21,494,047	21,494,047	19,784,129	19,242,503	90.80	18,270,154
C200T	19,588,861	19,588,861	17,953,109	17,510,470	90.50	16,566,068
SHEE	55,085,029	55,085,029	47,868,214	44,812,830	84.1	41,691,361
SHEEC	54,582,526	54,582,526	47,769,458	44,796,267	84.8	42,325,127

### Changes in AS patterns between ESCC tissue/matched normal tissue and two cell lines

**Fig. 1:**
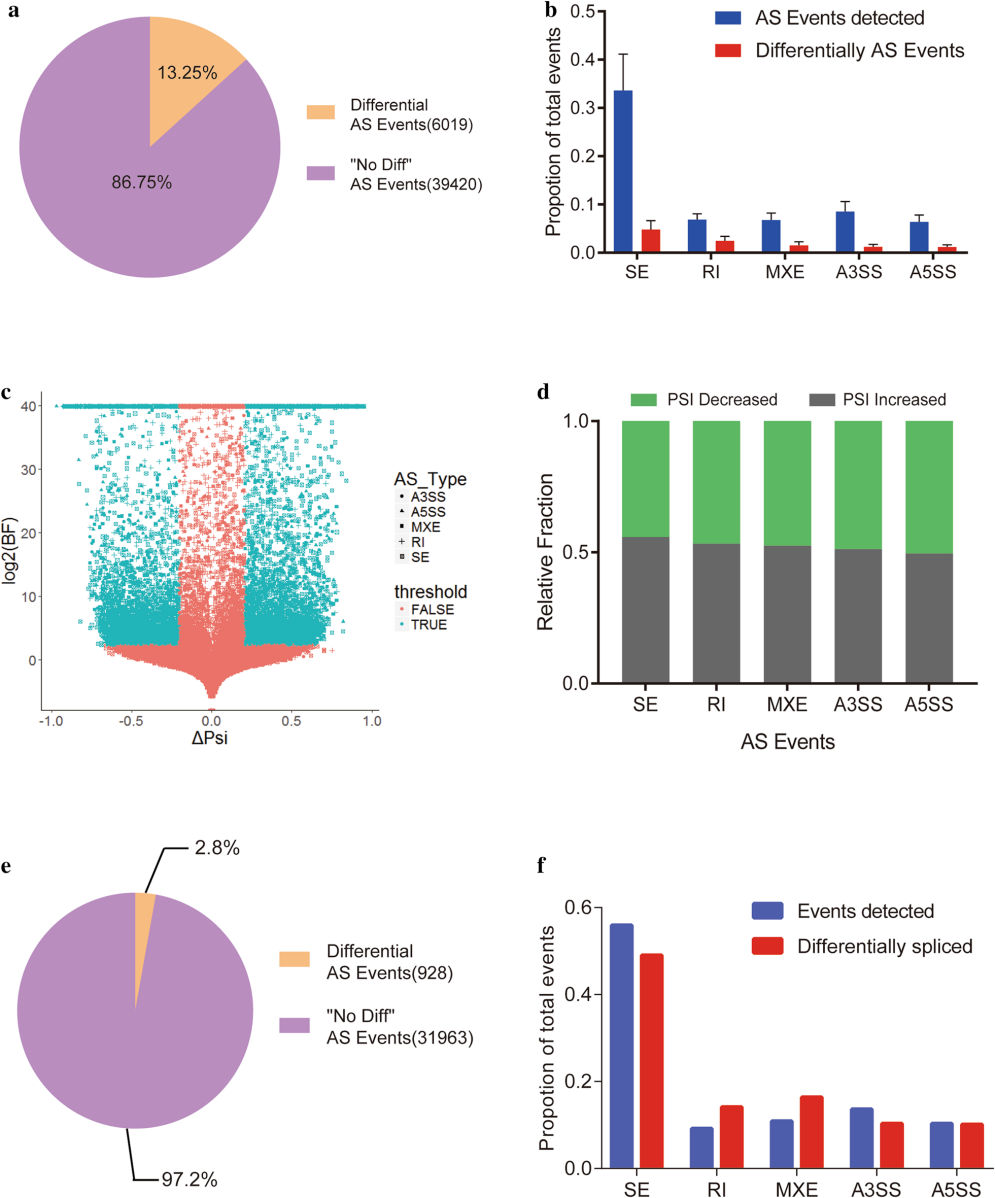
Distribution of RNA splicing events in ESCC after MISO analysis. **a** Percentages of differentially-spliced AS events with significance in total AS events found in ESCC clinical samples compared to the matched normal samples. **b** Plot depicts the fraction of events detected for each AS type (blue) compared to events with significantly different expression in ESCC tissues compared to matched normal tissues (red). **c** Scatter plot of all the AS events identified using MISO. The X-axis represents ΔΨ values, and the Y-axis represents log2 (BF) values. The shape of the dots indicates the type of AS event. Specifically, a hollow square indicates an SE event; cross indicates an RI event; solid square indicates an MXE event; triangle indicates an A5SS event; and circle indicates an A3SS event. Alternative splicing events with BF > 5 and ΔΨ > 0.2 are colored in blue. **d** Relative fraction of each AS event affected positively or negatively by ESCC. **e** Percentage of alternative splicing events showing significant changes in SHEE/SHEEC cell line association compared to detectable alternative splicing events showing no difference. **f** Plot depicts the fraction of events detected for each AS type (blue) compared to events with significantly different expressions in SHEE/SHEEC cell lines (red)

From the results of splicing analysis, Using MISO, a total of 45,439 AS events were detected. Of these, 6,019 AS events were significantly differently spliced in ESCC tissues as compared to normal tissues, with a cut-off of BF > 5, |ΔΨ|> 0.2. These significant AS events included a number of inclusion and exclusion reads > 10, accounting for 13.25% of the total number of AS events, suggesting a relative high splicing index in ESCC (Fig. 1a). After filtering out the AS events with inconsistent ΔΨ trends in the paired samples, there were 5,150 differential splicing events, including 2,324 exon skipping (SE) events, 1,014 intron retention (RI) events, and 693 mutually exclusive exon (MXE) events, 592 alternative 3’ splice site (A3SS) events, and 527 alternative 5’ splice site (A5SS) events. The AS results of ESCC tissue samples showed that the proportion of SE events that could be detected were the most abundant at about 33.6%, RI events at 6.9%, MXE 6.8%, A3SS 8.5%, and A5SS 6.4% of the total events. The percentage of SE events in differential splicing events was still the highest as 4.8%, while RI events comprising 2.5%, MXE events comprising 1.5%, A3SS events comprising 1.2%, and A5SS events comprising approximately 1.1%. Individual differences were detected during MISO analysis which were more obvious in the differential splicing events (Fig. 1b). From the volcano plot, it could be observed that the ΔΨ values and log2(BF) values in detectable AS events and the differentially spliced events were symmetrically distributed (Fig. 1c), Also, it could be seen from the and bar plot that the ΔΨ trend did not have a significant difference between AS event type (Fig. 1d).s

On the other hand, a total of 32,891 AS events were detected in the SHEE/SHEEC cell lines, of which a total of 928 AS events were significantly differentially spliced, accounting for approximately 2.8% of the total detectable AS events (Fig. 1e). The threshold for judging the differences in AS events in the SHEE/SHEEC cell lines was the same as applied in ESCC AS event filtration. In the detectable AS events, the proportion of SE was the largest, at 55.9%, with RI at 9.2%, MXE at 10.9%, A3SS accounting for 13.6%, and A5SS at 10.3%. Of the differential splicing events, the number of SE, RI, MXE, A3SS, and A5SS events were 455, 131, 152, 96, and 94, accounting for 49%, 14.1%, 16.4%, 10.3%, and 10.1% of the total number of differential splicing events, separately (Fig. 1f). The distribution of both total and differential AS is similar in ESCC tissues and ESCC cell lines.

### Re‐annotation of AS events in paired ESCC tissues

**Fig. 2:**
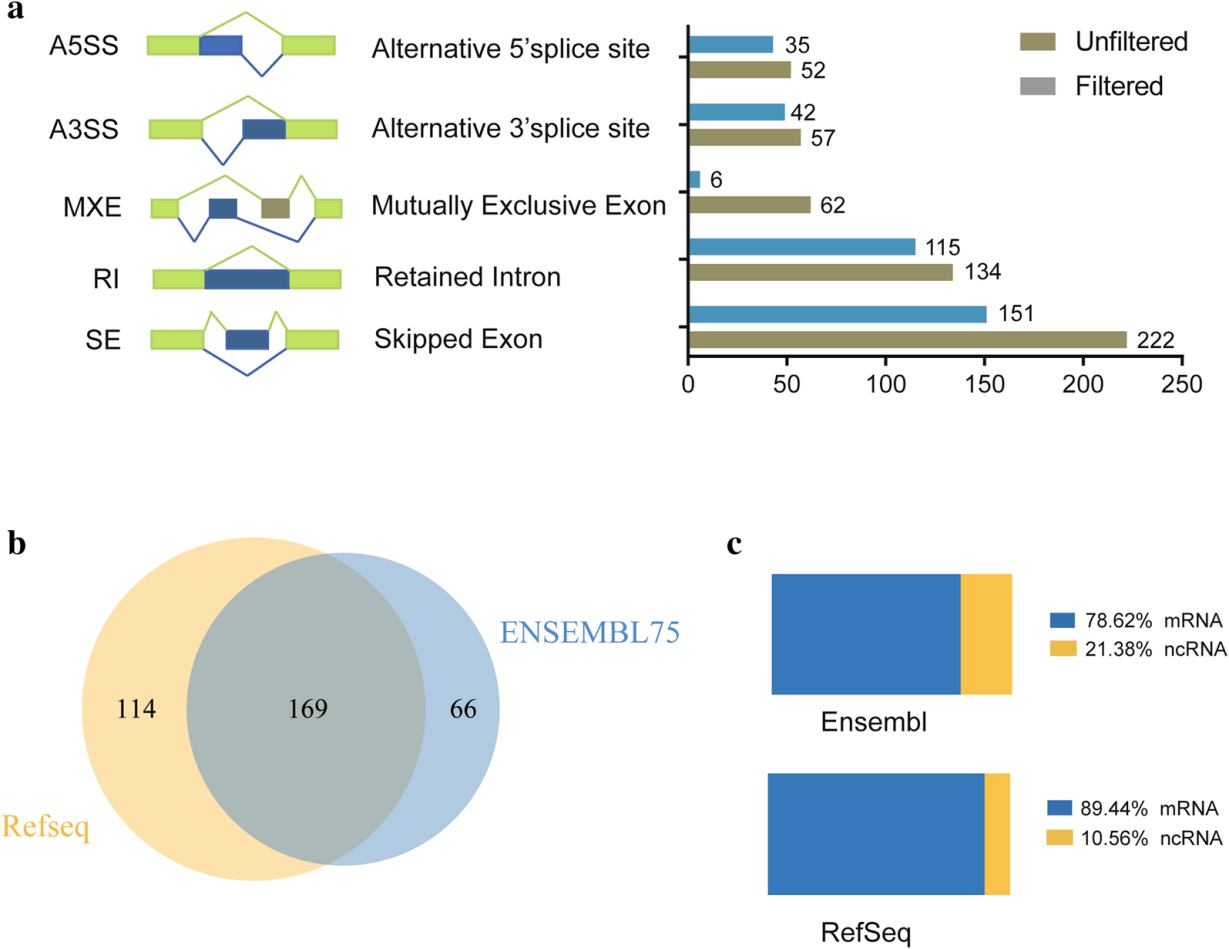
Distribution of RNA splicing events with significant differences in ESCC after re-annotation with Ensembl and RefSeq gene annotations. **a** Plot in the left panel indicates exon models of the types of AS assessed by MISO analysis. In the right panel, the different types of AS events before and after re-annotation were quantified with transcript annotations from Ensembl and RefSeq database, respectively. **b** A total of 283 and 235 splicing events with significantly altered PSI values were identified using transcript annotations from the RefSeq and Ensembl databases, respectively. In total, 169 splicing events were identified in both database transcript annotations. **c** Percentage of protein coding transcripts (blue) and non-coding transcripts (yellow) representing AS events after re-annotation analysis

**Fig. 3:**
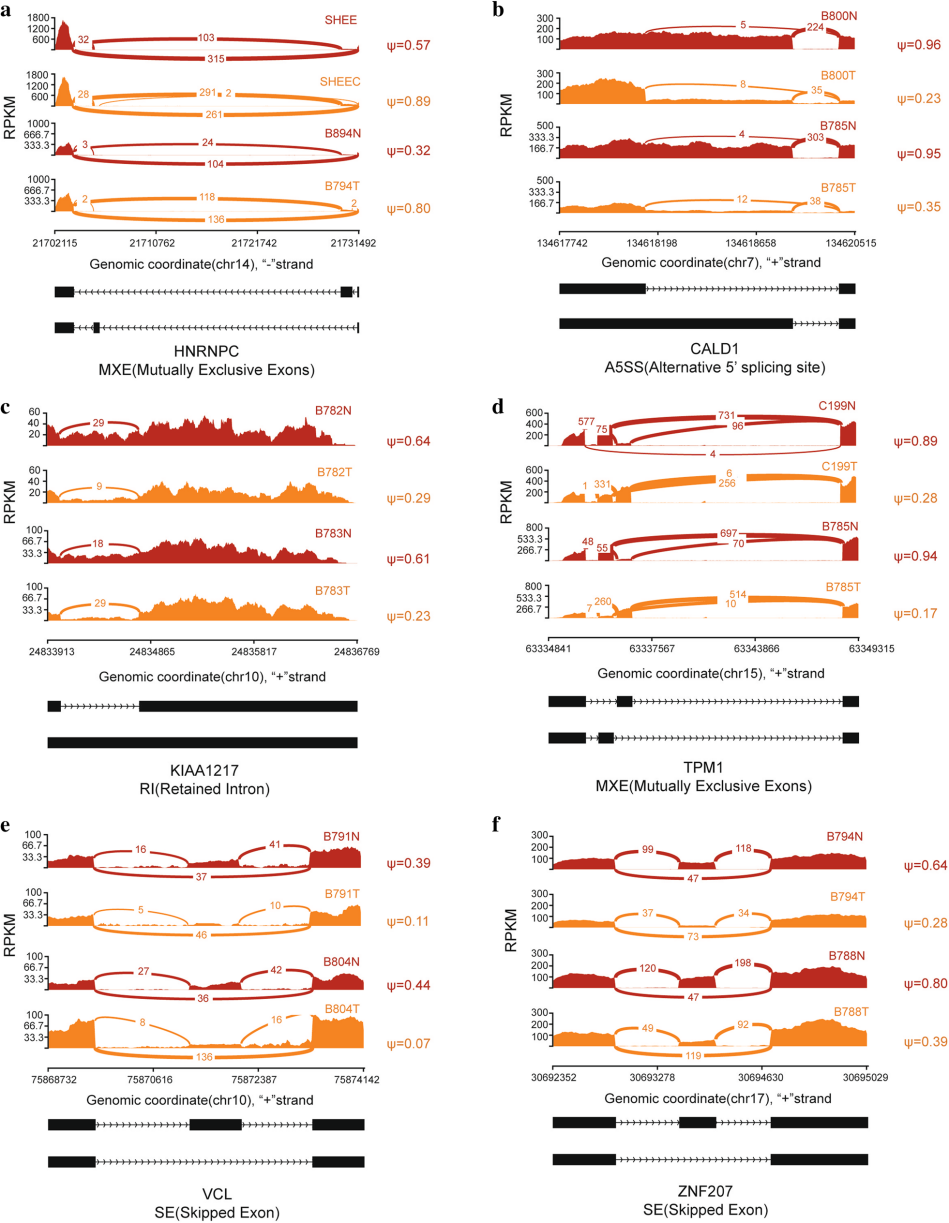
Sashimi plot of representative splicing changes in ESCC. Diagrams on the left show the read coverage of exons. Plots on the right show the Ψ values that occurred at least in two pair ESCC tissues or cell lines, as indicated by different colors. The AS model of this region is represented in each down panel. **a**−**f** AS model is shown for HNRNPC, CALD1, KIAA1217, TPM1, VCL and ZNF207, respectively. The curve indicates the splicing sites and the number in the curve suggests RNA-seq reads in this region

To identify the genes and transcripts representing the differential AS events and to filter out incorrect AS events, as described in the methods, we re-annotated all differentially splicing events found in at least 3 paired samples. The number of filtered significant AS events changed across the different categories of AS due to the re-annotation, but the event trend had minimal changes (Fig. 2a). For SE events, there were a total of 222 and 151 significant alternatively spliced events before and after re-annotation, reducing the number of AS events to approximately 68% of all events. There were a total of 134 RI significant AS events before re-annotation, and 115 after re-annotation, with a reduction of 14.2%. A total of 62 significant MXE events were identified before re-annotation and only 6 remained after re-annotation, reducing the total number to 9.7%. 57 significant A3SS events were found before re-annotation while 42 remained after re-annotation. 35 out of 52, 67.3%, significant A5SS events were identified after the re-annotation. Among these above results, SE events are the most prevalent in ESCC. The number of MXE events was reduced the most, because the two exons that should have been mutually exclusive in the MISO official MXE event annotation were observed to exist in the same transcript. Thus, a total of 349 significant AS events including five types were identified after re-annotation in 15 matched pairs of ESCC and normal tissue samples, of which transcripts/genes representing 169 AS events could be found both in the Ensembl and RefSeq annotations, whereas 114 could only be identified in the RefSeq annotations, and 66 AS events only in the Ensembl annotations (Fig. 2b). Through we found different annotations from these two popular databases, the majority of our annotation was found in both databases. We have also calculated the fractions of AS events representing protein-coding mRNA and ncRNA (non-coding RNA) (Fig. 2c). The percentage for ncRNA ranged from 10% to more than 20% in total events, suggesting alternative splicing is prevalent for ncRNA and its critical role in cancer [31]. The top 10 most prevalent splicing events that occurred in the paired samples are shown in Table 2. To better illustrate the different splicing patterns, HNRNPC, CALD1, KIAA1217, TPM1, VCL and ZNF207 were selected from the top genes and their splicing patterns are visualized by Sashimi plots (Fig. 3). These included representative examples for MXE, A5SS, RI and SE splicing types, which indicates the splicing diversity in ESCC.

**Table 2:** Summary of top 10 alternative splicing events in paired ESCC tissues

Splicing event	Gene name	Count of paired sample	Type
chr1:150,483,352:150,483,674: + @chr1:150,483,933:150,484,307: + @chr1:150,484,828:150,485,048: +	ECM1	14	SE
chr10:24,833,910–24,834,032: + @chr10:24,834,756–24,836,772: +	KIAA1217	13	RI
chr9:117,835,882:117,836,145:-@chr9:117,808,689:117,808,961:-@chr9:117,804,498:117,804,620:-	TNC	12	SE
chr15:63,334,838:63,335,142: + @chr15:63,335,905:63,336,030: + @chr15:63,336,226:63,336,351: + @chr15:63,349,184:63,349,317: +	TPM1	11	MXE
chr2:238,289,558:238,290,142:-@chr2:238,287,279:238,287,878:-@chr2:238,285,415:238,285,987:-	COL6A3	11	SE
chr22:31,495,731:31,495,882: + @chr22:31,496,871:31,497,035: + @chr22:31,500,302:31,500,610: +	SMTN	10	SE
chr16:15,808,766:15,808,938:-@chr16:15,802,660:15,802,698:-@chr16:15,796,992:15,797,980:-	MYH11	9	SE
chr17:30,692,349:30,692,506: + @chr17:30,693,684:30,693,776: + @chr17:30,694,791:30,695,033: +	ZNF207	9	SE
chr7:134,617,739:134,618,141|134,618,828: + @chr7:134,620,439:134,620,516: +	CALD1	9	A5SS
chr9:117,835,882:117,836,145:-@chr9:117,819,432:117,819,704:-@chr9:117,808,689:117,808,961:-	TNC	9	SE

### Overlapping AS events in ESCC tissues and SHEE/SHEEC cell lines

A total of 37 significant AS events were identified in both ESCC tissues and cell lines, 15 of which showed the same trend of ΔΨ values. Among these 15 AS events, there were 4 SE events, 7 RI events, 2 MXE events, and 2 A5SS events (Additional file 1: Table S1). Though the overlapping rate is quite low, we presume this might reflect the consensus and difference between clinical samples and cultured cell lines.

### Classification of AS events according to representative transcript types

After the re-annotation of the 349 AS events from ESCC tissues, 312 events represented at least one protein-coding transcript, which accounted for 89.4% of the total AS events. The remaining 37 AS events did not represent any protein-coding transcripts, including protein coding transcripts, lncRNAs, and microRNAs (Additional file 2: Table S2). Among the 235 AS events re-annotated by Ensembl transcript annotations, 200 events represented at least one protein-coding transcript, which accounted for 85.1% of the total AS events, and the remaining 35 AS events represented non-protein-coding transcripts. Of the 283 variable splicing events re-annotated by RefSeq transcript annotations, 266 events represented at least one protein-coding transcript, which accounted for 94% of the total AS events, and the remaining 17 AS events represented non-protein-coding transcripts. These results suggest that splicing of ncRNA transcripts could be just as prevalent as protein-coding transcripts.

### Functional enrichment revealed the potential roles of AS transcripts

**Fig. 4:**
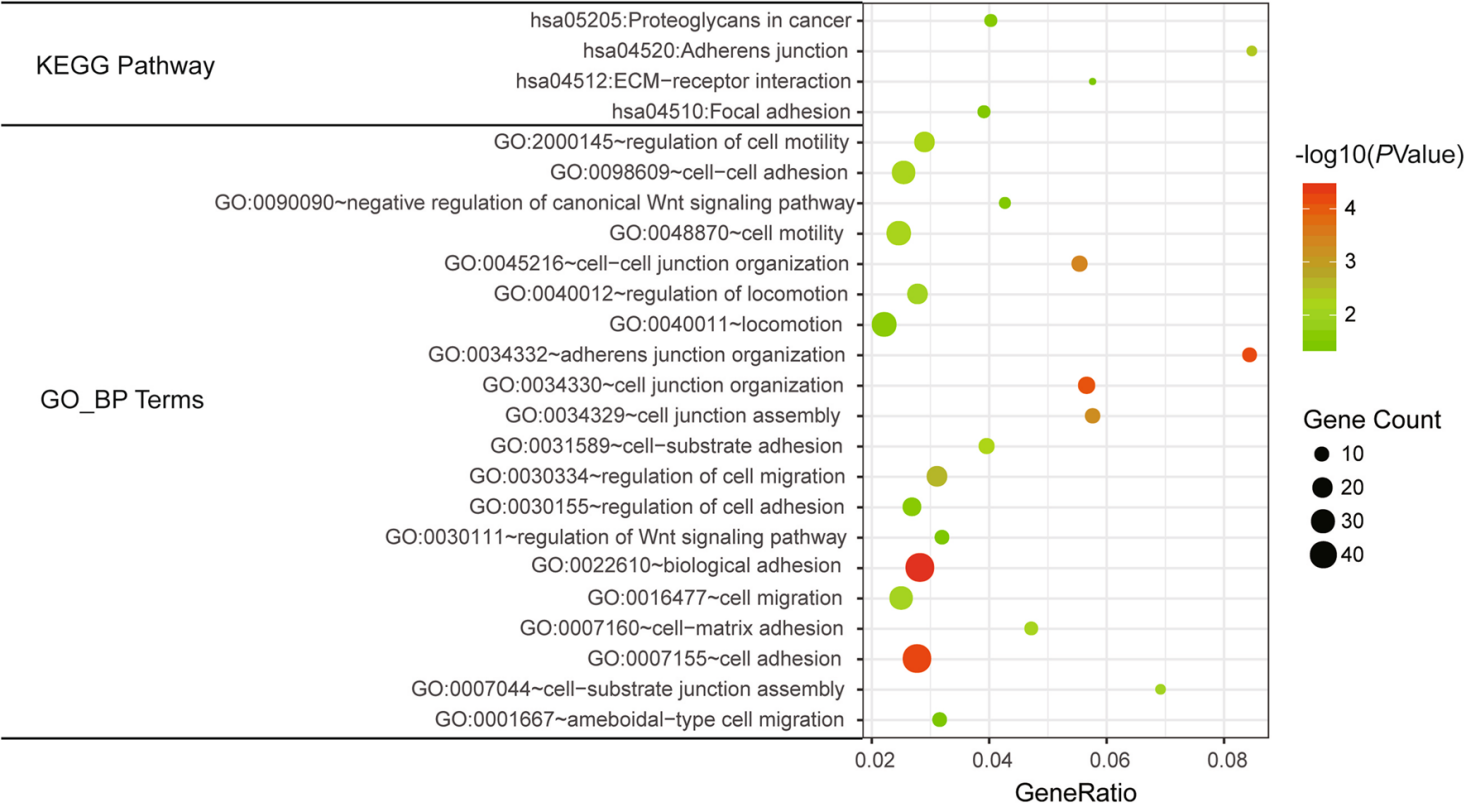
Dot plot of significant GO_BP terms and KEGG pathways using functional enrichment analyses with aberrant splicing event-related genes

To identify the biological processes and pathways affected by splicing abnormalities in ESCC, we performed the enrichment analyses. A total of 270 protein-coding genes were selected for functional and pathway enrichment analysis from the results of alternative splicing events after re-annotation of differential AS events with gene annotations from the Ensembl and RefSeq databases. A total of 234 genes were enriched in 543 biological processes. There were 382 biological processes satisfying the threshold of *P* < 0.05, and 39 genes were enriched in 12 pathways, of which 10 pathways satisfied *P* < 0.05. Interestingly, there were 4 KEGG pathways and 20 biological processes that potentially related to invasion and metastasis of cancer cells, such as adherens junction, ECM-receptor interaction, focal adhesion, regulation of cell mobility, regulation of cell migration and cell-matrix adhesion (Fig. 4). These results indicated that AS in ESCC might involve multiple cellular functions to promote tumor progression.

### Splicing regulatory network in ESCC

**Fig. 5:**
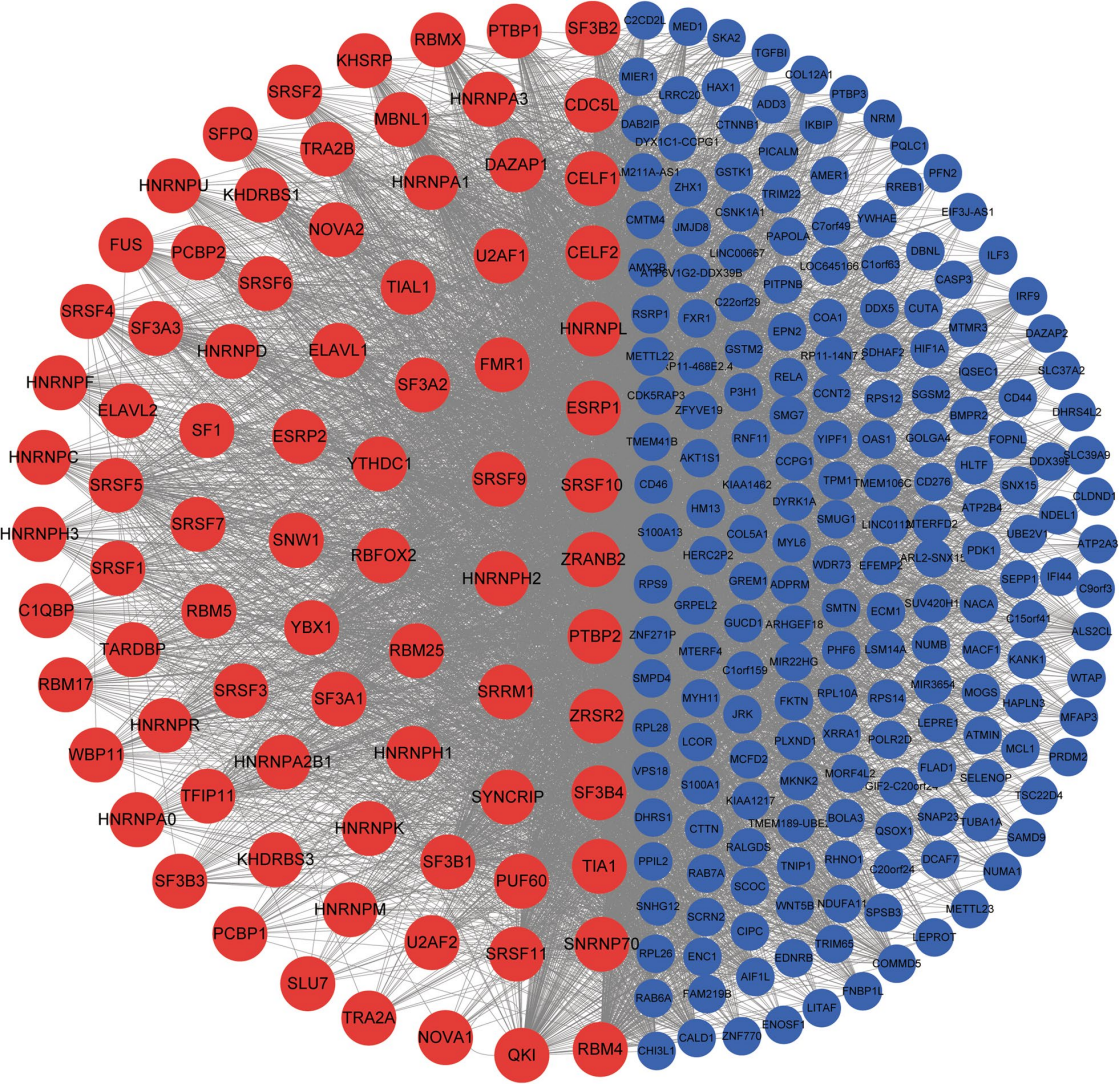
Regulatory network of splicing factor-AS events representing genes

To find the regulators of the splicing alterations in ESCC, we constructed a splicing regulatory network by calculating the FPKM values of splicing factors and PSI values of AS events. Filtered with a cut-off of *P* < 0.05 after Spearman correlation analysis, 5,999 splice factor-splicing event pairs and 5,511 splice factor-target gene pairs were chosen to build a splicing regulatory network. This network involved 81 splicing factors and 223 differential AS events (Fig. 5). Most of splicing factors could regulate dozens of target genes. On the other hand, one target gene could be processed by several splicing factors, suggesting the co-operation between these factors. Next, Splicing factors that were differentially expressed in ESCC and matched normal tissues were measured in two public microarray datasets (GSE53624 and GSE23400) and the RNA-seq dataset generated from our laboratory [16]. From these analyses, the splicing factor SF3B4 was significantly differentially expressed in ESCC with a 1.5 fold-change in three different datasets (Table 3).

**Table 3:** Summary of SF3B4 expression using three ESCC dataset

Dataset	Number of Samples		FoldChange	Pvalue	Qvalue	R packages
RNA-seq	15 pairs		1.42	0.197	0.458	DESeq
GSE53624	119 pairs		1.64	0	0	Siggenes
GSE23400	53 pairs		1.50	0	0	Siggenes

**Fig. 6:**
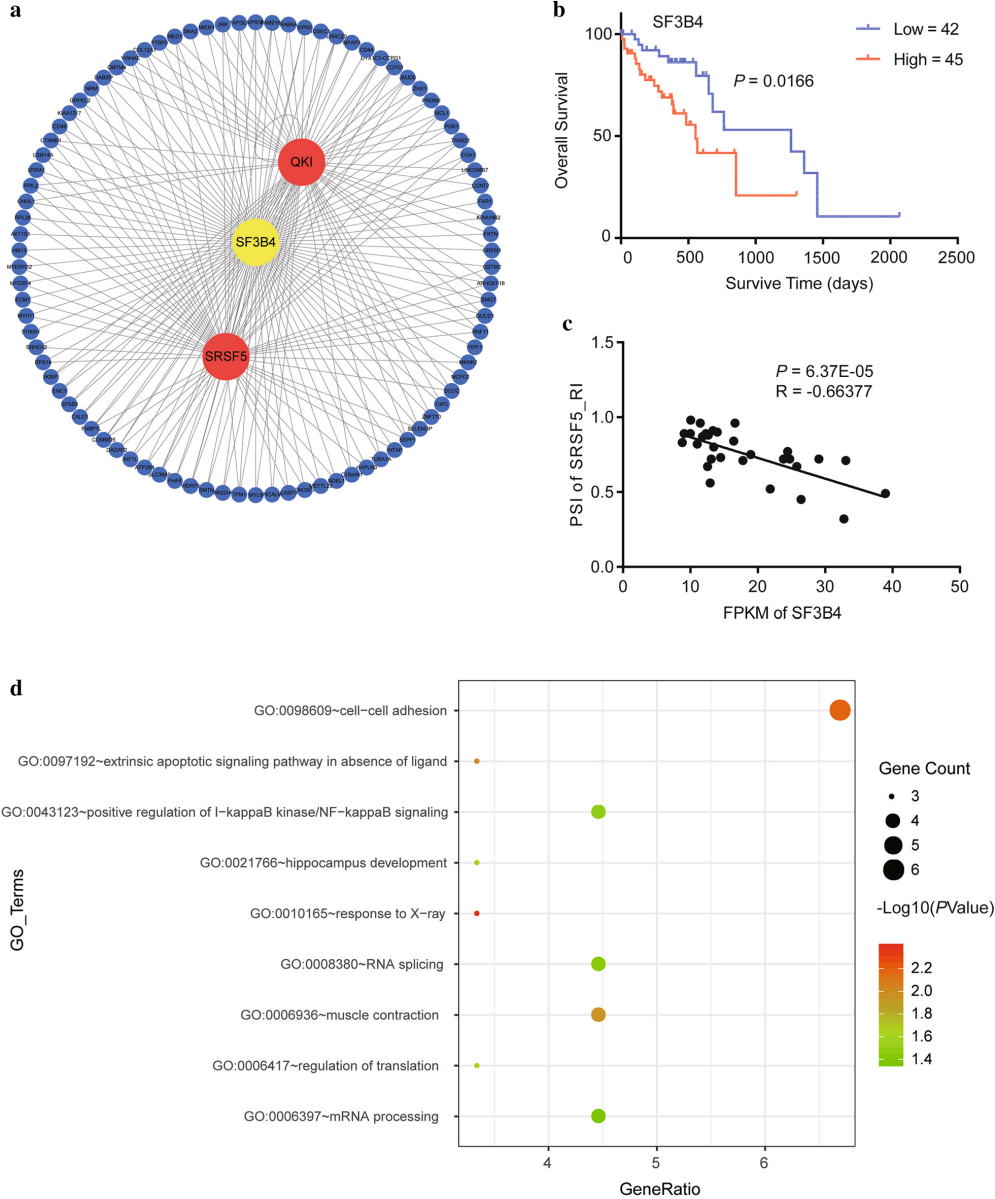
SF3B4-related regulatory network in ESCC. **a** SF3B4 splicing regulatory network. **b** K-M curve for SF3B4 in ESCC. **c** Scatter plot indicating correlation between SF3B4 expression and PSI value of the RI event of SRSF5. **d** Dot plot of GO_BP terms using alternatively-spliced SF3B4-targeted genes

For the splicing factors in the network, SF3B4 (splicing factor 3b subunit 4) caused us great interest. The splicing regulatory network for SF3B4 (splicing factor 3b subunit 4) and its target genes (with significant Spearman correlation) were also illustrated, which contained 92 target genes and 102 abnormal AS events (Fig. 6a), as some genes generated more than one AS event. Moreover, SF3B4 was found to be a survival-related gene in ESCC. In TCGA dataset, ESCC patients with a lower expression level of SF3B4 had a longer survival time (Fig. 6b). The PSI value of SRSF5’s RI event was found to have the smallest *p*-value with Spearman correlation analysis with the FPKM of SF3B4 (Fig. 6c). To understand the potential functions of SF3B4, functional enrichment analysis was also performed to identify the biological processes for SF3B4’s target genes, resulting in a dot plot of 9 significant biological processes, which involves cell-cell adhesion, NF-KB signaling and regulation of translation (Fig. 6d). Many evidences indicated AS are often linked to the occurrence of cancer driver mutations in genes encoding either core components or regulators of the splicing machinery [32]. However, we searched cBioPortal database (http://www.cbioportal.org/), but no mutation for SF3B4 in current TCGA ESCC clinical samples was found (data not shown).

### SF3B4 affects AS after its knockdown

To validate the regulatory role of SF3B4 on AS events in tumor samples, we have downloaded two RNA-seq datasets with SF3B4 knockdown, and calculated the inclusive levels of AS events with IncLevelDifference between control shRNA and SF3B4 knockdown (methods described in Additional file 3). From the 102 SF3B4 regulated AS events described above. We selected 36 AS events with top Spearman test P-value and top correlation (1 > |Rho| > 0.5), and named them as the top SF3B4 regulated AS events in ESCC samples. Subsequently, we tried to sieve out the overlapping results between the top SF3B4 regulated AS events in ESCC and significant AS events (FDR < 0.05) in either of the two validation datasets. Six overlapping AS events regulated by SF3B4 in tumor samples were finally verified, including 1 SE event (ATP2B4) and 5 RI events in 4 genes (SRSF5, PHF6, SNHG12, KIAA1217). It is interesting to point out that their Rho values are all negative, indicating SF3B4 represses intron retention in these transcripts. These results also suggest that SF3B4 could regulate AS in a pan-cancer manner, including in ESCC. The detailed information of the top 36 SF3B4 regulated AS events in ESCC are provided in Additional file 4: Table S3, with the 6 verified AS events indicated in the validated cell lines.

## Discussion

As an important step in post-transcriptional regulation, AS greatly contributes to proteome diversity, as well as cancer progression and development. As a common phenomenon in cancers, it has been reported that an endogenous soluble form of VEGFR-2, a product of alternative splicing, is present in humans and mice [33]. Our previous research found that endogenous soluble VEGFR-2 is down-regulated in ESCC, but its high expression is an independent prognostic factor for poor survival [33]. We identified a novel splice variant of LOXL2 (Lysyl oxidase-like 2), named LOXL2Δ72, which lacks 72 nucleotides encoding 24 amino acids [34]. Though LOXL2Δ72 has dramatically reduced enzymatic activity, it promotes greater cell migration and invasion than its wild type counterpart [34]. However, alternative splicing studies in ESCC remain at a low-throughput level, and analyses of AS events in ESCC on a genome-wide scale are still rare. Although aberrant splicing events in ESCA samples have been found and related to overall survival, detailed splicing patterns of ESCC are still unknown, and splicing-mode changes between ESCC and matched normal tissues have not been characterized [35]. In this paper, previously published next-generation sequencing data was used to study AS in ESCC. Because our RNA-seq data was generated from ESCC tissue and matched normal tissue of the same patient, our data is suitable for MISO analyses and splicing-mode change characterization. There are RNA-seq data generated from a pair of cell line samples, an immortalized human esophageal epithelial cell line (SHEE) and its malignant transformed cell line (SHEEC), which was also used as a validating dataset. This comparison reflects the consistence and difference between cancer tissues and cultured cells while considering their AS pattern.

In total, 6,019 significant AS events were identified in 15 pairs of ESCC tissues. It is also noteworthy that a large proportion of the AS events were found to only have significant differences in a small number of paired samples, which suggests that AS differences in many genes might have a similar impact on the development of ESCC. The proportion of AS events with ΔΨ > 0 and ΔΨ < 0 in ESCC tends to be consistent, which indicates that the current AS classification system cannot promise all the true AS types, and the real AS modes may be more complicated. We have noticed that the AS annotations in MISO are based on existing transcripts, so alternative splicing analysis can not discover new transcripts, but unknown AS regulatory mechanisms based on known genes and transcripts could be revealed by the MISO analysis. Even so, there are many problems with the official MISO AS annotations, in which several exons in mutually exclusive exon events are found present in the same transcript, making the accuracy of the annotation confusing. In this study, transcriptional annotations from Ensembl and RefSeq database were used to re-annotate the previously analyzed AS events and to remove incorrect splicing events to make the subsequent analysis more accurate.

Many laboratories have reported some clues on the mechanism of alternative splicing affecting cancers. For example, the normal transcript for CD44 was expressed in normal gastric tissue, and the upregulation of its splice variant is associated with invasion and metastasis of gastric tumors [36]. Higher expression of CD44 variant 6 has been reported in gastric tumor with lymph node metastasis, suggesting that CD44 variant 6 plays a role in the metastasis of gastric cancer [37]. BP1, a splicing isoform of DLX4, is highly expressed in endometrial cancer, which correlates with patient poor prognosis, cancer pathological grade, tumor invasion and metastasis [38]. For the first time, from the perspective of systems biology, we show the biological processes and KEGG pathways affected by alternative splicing in ESCC development. By functional enrichment analyses, multiple GO terms and pathways are observed in relation to cell junction and cell migration, indicating that altered splicing may promote invasion and metastasis in ESCC.

For the top differential AS genes generated from ESCC in this study, many of them have been reported to play an important role in tumor progression. HNRNPC is a typical splicing factor, and its regulation by alternative splicing has not been reported. Alternative splicing of ZNF207 has been reported to be regulated by SRSF11 [39], but its AS pattern has not been reported. VCL encodes an F-actin-binding cytoskeletal protein, compared to VCL-001, VCL-201 contains an extra exon named exon 19. The skipped exon 19 is reported to be increasingly preferred in colon tumor cells compared to normal mucosa [40], which is consistent with its Sashimi plot model shown in this study. CALD1 is a calmodulin- and actin-binding protein with regulatory functions, and has been reported to play an important role in cell movement. In colon cancer tissues, the longest CALD1 transcript is reported to be reduced, with the increase of its shorter splicing isoform [40]. However, Sashimi plot results in this study show that there is an alternative 5’ splice site event in CALD1, which also involves a relatively down-regulated expression of its longest isoform. Intron retention of KIAA1217 has been found in non-small cell lung cancer, but RT-PCR results do not support this result [41]. Our results indicate that there is an RI event for KIAA1217, but the retained intron tends to be included in matched normal tissues. Regarding TPM1, it has been reported that exon 6 of TPM1 has two types, TPM1-6A and TPM1-6B, but are the same 76 bp size. In bladder cancer and prostate cancer, TPM1 exon 6A and 6B are reported to have opposite splicing trends [42]. We also show there is an MXE event in TPM1s, and both the mutually exclusive exons are 76 bp long. Though our top AS genes are generated from paired ESCC tissues and cell lines, they need further verification, both in clinical samples and cultured cell lines.

Splicing factors, which participate in the ribonucleoprotein (RNP) complexes that play key roles in AS regulation often drive different AS patterns in a tissue- and cell-type-specific manner, both in normal and cancerous conditions [43]. A key role for SF3B4 in diseases in regards to its splicing function has been revealed in recent years. A heterozygosity for SF3B4 mutations is found in Rodriguez syndrome, which leads to reduced SF3B4 synthesis and defects in mRNA splicing, primarily exon skipping [44]. Overexpressing SF3B4 resulted in a mis-splicing of Kruppel-like factor 4 (KLF4), a tumor suppressor-encoding gene, into a non-functional transcript in cancer cells, and thus promoting tumorigenesis in HCC [45].

But the relationship between SF3B4 and ESCC has not been identified. In this paper, we found that SF3B4 is up-regulated in ESCC, which is similar to the results of an HCC study, in which SF3B4 is an up-regulated diagnostic marker [46]. Moreover, according to the log-rank (Mantel-Cox) test, SF3B4 can be identified as a prognostic marker of ESCC. Consistently, a new paper found over-expression of SF3B4 may play a crucial role in the lymphatic progression of ESCC, during the submission of our study [47]. Spearman correlation analysis was utilized to calculate the correlation between the degree of splicing events and the expressions of splicing factors in ESCC, according to a previous method [48]. SF3B4 was also found to play an important role in regulating 92 protein coding genes and their aberrant splicing events in ESCC. Functional enrichment analysis indicates that SF3B4’s downstream targets may play a role in cell-cell junction and nuclear factor kB (NF-kB) processes.

To identify the target genes of SF3B4 in tumors, significant AS events were investigated in SF3B4 knockdown cells. Six AS events regulated by SF3B4 in tumor samples overlapped with the top SF3B4 regulated AS events in ESCC. The oncogenic role of SRSF5 [49], PHF6 [50], SNHG12 [51] and KIAA1217 [52] have been reported in different tumors, but we firstly discovered that intron retention in these genes are repressed by SF3B4. Retained intron (RI) repression potentially leads to productive transcripts, because retained introns usually produce premature termination codons, which may generate unproductive, unstable transcripts or truncated proteins [53]. These results reveal that SF3B4 regulates the expression pattern of many cancer-associated genes through AS mechanisms. However, it is still necessary to verify their regulatory mechanism and subsequent biological effects through validation experiments.

## Conclusions

In this study, AS events were analyzed from RNA-seq data from matched normal and ESCC tissue samples\ pairs and two cell lines where 13.25% significant differential AS events were identified, which are involved in cell junction and migration. The splicing factor SF3B4 is a potential prognostic factor and a splicing regulator in ESCC. In summary, we identified thousands of significant differential AS events which might be related to the development of ESCC.

## Supplementary Information

**Additional file 1: Table S1.** Summary of alternative splicing events with significant difference in ESCC tissues & cell lines.

**Additional file 2: Table S2.** Alternative splicing events representing non-coding RNA.

**Additional file 3.** Materials and methods.

**Additional file 4: Table S3.** SF3B4 regulated alternative splicing events tested in SF3B4 shRNA knockdown cell lines.

## Data Availability

The datasets used and/or analyzed during the current study are available from the corresponding author on reasonable request.
