# Supplementary material for: Alterations of RNA splicing patterns in esophagus squamous cell carcinoma
Source: Cell Biosci. 2021 Feb 9;11:36. doi: 10.1186/s13578-021-00546-z (PMC7871539; doi:10.1186/s13578-021-00546-z)
Supplement: Supplementary file 1 — Additional file 1: Table S1. Summary of alternative splicing events with significant difference in ESCC tissues & cell lines. [file 13578_2021_546_MOESM1_ESM.docx]

**Additional file 1: Table S1. Summary of alternative splicing events with significant difference in ESCC tissues & cell lines**

| AS_Type | Event | Sample_ID | Gene_Name | Transcript_ID |
| --- | --- | --- | --- | --- |
| SE | chr5:150414563:150414628:-@chr5:150411848:150411944:-@chr5:150409504:150410308:- | B782  B783  B791  B794  B799  SHEE/SHEEC | TNIP1 | NM_001258454 NM_001252390 NM_006058 NM_001252385 NM_001258455 NM_001252386 NM_001252391 NM_001258456 |
|  | chr9:128469250:128469513:-@chr9:128432097:128432186:-@chr9:128419930:128420078:- | B783  B791  C199  SHEE/SHEEC | MAPKAP1 | NM_024117 NM_001006618 ENST00000394063 NM_001006619 NM_001006617 NM_001006621 ENST00000265960 NM_001006620 |
|  | chr1:161129254:161129468:+  @chr1:161130156:161130296:+  @chr1:161130410:161131030:+ | B804  B782  C199 SHEE/SHEEC | USP21 | NM_001014443 NM_001319848 NM_012475 ENST00000368002 |
|  | chr22:42998776:42999166:-@chr22:42997976:42998113:-@chr22:42995704:42995799:- | C199  B782  B788 SHEE/SHEEC | POLDIP3 | NM_032311 NR_103820 NM_001278657 NM_178136 |
| RI | chr14:24633272-24633343:+  @chr14:24633823-24634164:+ | C200  B791  B798  SHEE/SHEEC | IRF9/ ENSG00000259529 | ENST00000396864 ENST00000558468 ENST00000561342 ENST00000557894 NM_006084 |
|  | chr3:46717892-46717735:-@chr3:46717466-46716056:- | C199  B785  C200  B791 SHEE/SHEEC | ALS2CL | ENST00000486301 |
|  | chr1:180165397-180165734:+  @chr1:180166468-180167169:+ | B786  B791  B782  B804 SHEE/SHEEC | QSOX1 | ENST00000367600 NM_001004128 |
|  | chr1:25571792-25571641:-@chr1:25570715-25570041:- | B786,  B800,  B797,  B788, SHEE/SHEEC | RSRP1 | ENST00000568254 NR_135787 NR_135781 NR_135144 ENST00000473314 |
|  | chr14:20942631-20942734:+  @chr14:20942932-20943107:+ | B804  B782  B788  B786 SHEE/SHEEC | PNP | ENST00000553591 ENST00000361505 NM_000270 |
|  | chr6:33386065-33385864:-@chr6:33385472-33385258:- | B797  B788  B800  B801 SHEE/SHEEC | CUTA | NM_001014433 |
|  | chr7:142961641-142961769:+  @chr7:142962085-142962185:+ | B786  B791  B785  C199 SHEE/SHEEC | GSTK1 | ENST00000436038 ENST00000442394 NM_001143680 ENST00000409500 ENST00000358406 NM_015917 |
| MXE | chr14:21731470:21731495:-@chr14:21730760:21730927:-@chr14:21702928:21702990:-@chr14:21702112:21702388:- | B794  B786  C199  B791 SHEE/SHEEC | HNRNPC | ENST00000554891 ENST00000420743 |
|  | chr14:21731470:21731495:-@chr14:21730760:21730927:-@chr14:21704532:21704619:-@chr14:21702112:21702388:- | B794  B791  C199  B786 SHEE/SHEEC | HNRNPC | ENST00000556226 ENST00000555176 ENST00000420743 ENST00000555137 |
| A5SS | chr6:33386065:33385646\|33385864:-@chr6:33385258:33385472:- | B801  B782  B788  B791 SHEE/SHEEC | CUTA | NM_001014838 NM_001014433 |
|  | chr7:134855578:134855151\|134855404:-@chr7:134853501:134853812:- | B799  B791  B783 SHEE/SHEEC | C7orf49 | NM_001305629 NM_001243749 NM_001305630 NM_001243751 |
