# Supplementary material for: Alterations of RNA splicing patterns in esophagus squamous cell carcinoma
Source: Cell Biosci. 2021 Feb 9;11:36. doi: 10.1186/s13578-021-00546-z (PMC7871539; doi:10.1186/s13578-021-00546-z)
Supplement: Supplementary file 2 — Additional file 2: Table S2. Alternative splicing events representing non-coding RNA. [file 13578_2021_546_MOESM2_ESM.docx]

**Additional file 2: Table S2. Alternative splicing events representing non-coding RNA**

| Event | Gene_Symbol | Transcript_ID | Type |
| --- | --- | --- | --- |
| chr1:148932786:148932920:+@chr1:148933291:148933368:+@chr1:148951245:148951595:+ | RP11-14N7.2 | ENST00000539543 | SE |
| chr20:34328746:34328809:-@chr20:34328447:34328519:-@chr20:34327025:34327469:- | RBM39 | ENST00000463098 | SE |
| chr3:46713523-46713374:-@chr3:46713258-46712978:- | ALS2CL | ENST00000473484 | RI |
| chr3:46717892-46717735:-@chr3:46717466-46716056:- | ALS2CL | ENST00000486301 | RI |
| chr9:33443456-33443177:-@chr9:33442968-33442850:- | AQP3 | ENST00000473153 | RI |
| chr15:37100525-37100690:+@chr15:37101105-37102449:+ | C15orf41 | ENST00000565792 | RI |
| chr1:25571792-25571641:-@chr1:25570715-25570041:- | C1orf63 | ENST00000473314 | RI |
| chr1:25571792-25571641:-@chr1:25570715-25570041:- | C1orf63 | ENST00000568254 | RI |
| chr15:74005275-74005404:+@chr15:74005631-74006855:+ | CD276 | ENST00000559073 | RI |
| chr17:61666362-61666599:+@chr17:61671565-61671628:+ | DCAF7 | ENST00000582103 | RI |
| chr17:18002331-18002391:+@chr17:18002947-18003749:+ | DRG2 | ENST00000582314 | RI |
| chr11:65634550-65634316:-@chr11:65634183-65633912:- | EFEMP2 | ENST00000531972 | RI |
| chr17:16342641-16342728:+@chr17:16342842-16343017:+ | FAM211A-AS1 | ENST00000472367 | RI |
| chr17:16342641-16342728:+@chr17:16342842-16343017:+ | FAM211A-AS1 | ENST00000478103 | RI |
| chr17:16342641-16343017:+@chr17:16343499-16343567:+ | FAM211A-AS1 | ENST00000480811 | RI |
| chr5:179044111-179043870:-@chr5:179043219-179043127:- | HNRNPH1 | ENST00000521720 | RI |
| chr17:1617308-1616997:-@chr17:1615693-1614805:- | MIR22HG | ENST00000334146 | RI |
| chr11:62342380-62342149:-@chr11:62340214-62340056:- | MIR3654 | ENST00000526409 | RI |
| chr17:72741445-72741629:+@chr17:72742574-72743474:+ | RAB37 | ENST00000481224 | RI |
| chr9:135977526-135977325:-@chr9:135977149-135973107:- | RALGDS | ENST00000482648 | RI |
| chr14:69925080-69925446:+@chr14:69928414-69929098:+ | SLC39A9 | ENST00000555840 | RI |
| chr1:28907158-28907072:-@chr1:28906493-28906045:- | SNHG12 | ENST00000470977 | RI |
| chr14:70235899-70235968:+@chr14:70236228-70237257:+ | SRSF5 | ENST00000555547 | RI |
| chr14:70237911-70238261:+@chr14:70238634-70238722:+ | SRSF5 | ENST00000556647 | RI |
| chr11:9305140-9304918:-@chr11:9302588-9302201:- | TMEM41B | ENST00000299596 | RI |
| chr15:85189579-85189204:-@chr15:85189067-85188702:- | WDR73 | ENST00000559994 | RI |
| chr15:23285705:23285752:-@chr15:23283153\|23283416:23282281:- | HERC2P2 | ENST00000560464 | A3SS |
| chr15:23285705:23285752:-@chr15:23283153\|23283416:23282281:- | HERC2P2 | ENST00000454333 | A3SS |
| chr16:16427504:16427741:+@chr16:16429718\|16429909:16429994:+ | NPIPA7 | ENST00000537112 | A3SS |
| chr18:5238099:5239048:+@chr18:5240186\|5241698:5241811:+ | LINC00667 | ENST00000582008 | A3SS |
| chr20:30135185:30135409:+@chr20:30136183\|30136480:30136596:+ | HM13 | ENST00000487964 | A3SS |
| chr6:31509727:31509925:-@chr6:31508311\|31508441:31508099:- | ATP6V1G2-DDX39B | ENST00000376185 | A3SS |
| chr12:120636803:120636657\|120636698:-@chr12:120636357:120636573:- | RPLP0 | ENST00000547173 | A5SS |
| chr12:120636803:120636657\|120636698:-@chr12:120636357:120636573:- | RPLP0 | ENST00000551258 | A5SS |
| chr1:148932786:148932920:+@chr1:148933291:148933368:+@chr1:148951245:148951595:+ | LOC645166 | NR_027355.2 | SE |
| chr15:44828999:44829098:-@chr15:44828241:44828556:-@chr15:44826703:44827672:- | EIF3J-AS1 | NR_034171.1 | SE |
| chr18:32870236:32870355:+@chr18:32870974:32871196:+@chr18:32885940:32890730:+ | ZNF271P | NR_024565.1 | SE |
| chr2:110744557:110744767:+@chr2:110745138:110746379:+@chr2:110751978:110752858:+ | LINC01123 | NR_046110.1 | SE |
| chr2:110744557:110744767:+@chr2:110745138:110746379:+@chr2:110751978:110752858:+ | LINC01123 | NR_046111.1 | SE |
| chr7:43679048:43679280:-@chr7:43670783:43671381:-@chr7:43648055:43649240:- | COA1 | NR_135580.1 | SE |
| chr1:148932786:148932920:+@chr1:148933291:148933368:+@chr1:148951245:148951595:+ | LOC645166 | NR_027356.2 | SE |
| chr18:32870236:32870355:+@chr18:32870974:32871196:+@chr18:32885940:32890730:+ | ZNF271P | NR_024566.1 | SE |
| chr2:110744557:110744767:+@chr2:110745138:110746379:+@chr2:110751978:110752858:+ | LINC01123 | NR_046112.1 | SE |
| chr7:43679048:43679280:-@chr7:43670783:43671381:-@chr7:43648055:43649240:- | COA1 | NR_135581.1 | SE |
| chr7:43679048:43679280:-@chr7:43670783:43671381:-@chr7:43648055:43649240:- | COA1 | NR_135583.1 | SE |
| chr7:43679048:43679280:-@chr7:43670783:43671381:-@chr7:43648055:43649240:- | COA1 | NR_135582.1 | SE |
| chr1:25571792-25571641:-@chr1:25570715-25570041:- | RSRP1 | NR_135144.1 | RI |
| chr1:25571792-25571641:-@chr1:25570715-25570041:- | RSRP1 | NR_135781.1 | RI |
| chr1:25571792-25571641:-@chr1:25570715-25570041:- | RSRP1 | NR_135787.1 | RI |
| chr11:65634550-65634316:-@chr11:65634183-65633912:- | EFEMP2 | NR_037718.1 | RI |
| chr11:9305140-9304918:-@chr11:9302588-9302201:- | TMEM41B | NR_028491.2 | RI |
| chr17:1617308-1616997:-@chr17:1615693-1614798:- | MIR22HG | NR_028505.1 | RI |
| chr17:1617308-1616997:-@chr17:1615693-1614798:- | MIR22HG | NR_028504.1 | RI |
| chr17:1617308-1616997:-@chr17:1616189-1614798:- | MIR22HG | NR_028503.1 | RI |
| chr17:16342641-16342728:+@chr17:16342842-16343017:+ | LRRC75A-AS1 | NR_027169.1 | RI |
| chr17:16342641-16342728:+@chr17:16342842-16343017:+ | LRRC75A-AS1 | NR_027163.1 | RI |
| chr17:16342641-16342728:+@chr17:16342842-16343017:+ | LRRC75A-AS1 | NR_045026.1 | RI |
| chr6:31509727:31509925:-@chr6:31508311\|31508441:31508099:- | ATP6V1G2-DDX39B | NR_037853.1 | A3SS |
| chr18:5238099:5239048:+@chr18:5240186\|5241698:5241811:+ | LINC00667 | NR_015389.1 | A3SS |
| chr17:1616997:1617308:-@chr17:1615693\|1616189:1614798:- | MIR22HG | NR_028503.1 | A3SS |
| chr17:1616997:1617308:-@chr17:1615693\|1616189:1614798:- | MIR22HG | NR_028504.1 | A3SS |
| chr17:1616997:1617308:-@chr17:1615693\|1616189:1614798:- | MIR22HG | NR_028505.1 | A3SS |
| chr16:16427504:16427741:+@chr16:16429718\|16429909:16429994:+ | PKD1P1 | NR_036447.1 | A3SS |
