# Supplementary material for: Alterations of RNA splicing patterns in esophagus squamous cell carcinoma
Source: Cell Biosci. 2021 Feb 9;11:36. doi: 10.1186/s13578-021-00546-z (PMC7871539; doi:10.1186/s13578-021-00546-z)
Supplement: Supplementary file 3 — Additional file 3. Materials and methods. [file 13578_2021_546_MOESM3_ESM.docx]

**Supplementary Materials and Methods**

**SF3B4 regulated AS events verification**

# Two RNA-seq dataset used to verify SF3B4 regulated AS events in tumor samples were downloaded from the ENCODE portal [[1](#_ENREF_1)] with the following 4 identifiers for 8 samples: [ENCSR344XID](https://www.encodeproject.org/experiments/ENCSR344XID/) (control non-targeting shRNA, human leukemic cell line k562), [ENCSR081XRA](https://www.encodeproject.org/experiments/ENCSR081XRA/) (shRNA knockdown against SF3B4, K562), [ENCSR603TCV](https://www.encodeproject.org/experiments/ENCSR603TCV/) (control non-targeting shRNA, human liver carcinoma cell line HepG2), [ENCSR148MQK](https://www.encodeproject.org/experiments/ENCSR148MQK/) (shRNA knockdown against SF3B4, HepG2). The software rMATS v4.1.0 [[2](#_ENREF_2)] was applied with python v3.6 to perform AS analysis with the comparison of two control vs two SF3B4 knockdown samples. The inclusive levels of AS events IncLevelDifference was analyzed, which presents as the difference in average psi values between reference and alternative allele samples. The significant differential AS events from these validation dataset were compared the differential AS events in ESCC from this study.

**References**

1. Davis CA, Hitz BC, Sloan CA, Chan ET, Davidson JM, Gabdank I, Hilton JA, Jain K, Baymuradov UK, Narayanan AK et al: The Encyclopedia of DNA elements (ENCODE): data portal update. Nucleic acids research 2018, 46(D1):D794-d801.
2. Shen S, Park JW, Lu ZX: rMATS: robust and flexible detection of differential alternative splicing from replicate RNA-Seq data. 2014, 111(51):E5593-5601.
