# Supplementary material for: Alterations of RNA splicing patterns in esophagus squamous cell carcinoma
Source: Cell Biosci. 2021 Feb 9;11:36. doi: 10.1186/s13578-021-00546-z (PMC7871539; doi:10.1186/s13578-021-00546-z)
Supplement: Supplementary file 4 — Additional file 4: Table S3. SF3B4 regulated alternative splicing events tested in SF3B4 shRNA knockdown cell lines. [file 13578_2021_546_MOESM4_ESM.docx]

**Supplementary Table 3. SF3B4 regulated alternative splicing events tested in SF3B4 shRNA knockdown cell lines.**

| **Event** | **Gene_name** | **Type** | **corr_Rho** | **cell_line_tested** | **FDR_tested** |
| --- | --- | --- | --- | --- | --- |
| chr14:70235899-70235968:+@chr14:70236228-70237257:+ | SRSF5 | RI | -0.66 | HepG2 | 7.21E-09 |
| chr1:203702351:203702458\|203702528:+@chr1:203708674:203713209:+ | ATP2B4 | A5SS | -0.66 |  |  |
| chr7:43679048:43679280:-@chr7:43670783:43671381:-@chr7:43648055:43649240:- | COA1 | SE | 0.64 |  |  |
| chr1:203696523:203696699:+@chr1:203702351:203702528:+@chr1:203708674:203713209:+ | ATP2B4 | SE | -0.63 | k562 | 0.01021 |
| chr15:63334838:63335142:+@chr15:63335905:63336030:+@chr15:63336226:63336351:+@chr15:63349184:63349317:+ | TPM1 | MXE | -0.62 |  |  |
| chr1:54325729:54325826:-@chr1:54320674:54320724:-@chr1:54317392:54317943:- | YIPF1 | SE | -0.59 |  |  |
| chr16:733167:733234:-@chr16:732953:733087:-@chr16:731667:732874:- | JMJD8 | SE | -0.59 |  |  |
| chr3:180687946:180688146:+@chr3:180688863:180688943:+@chr3:180693101:180693192:+ | FXR1 | SE | -0.57 |  |  |
| chr15:63334838:63335142:+@chr15:63335905\|63336226:63336351:+ | TPM1 | A3SS | -0.57 |  |  |
| chrX:133559231-133559360:+@chrX:133559691-133562819:+ | PHF6 | RI | -0.57 | HepG2 | 0.040546 |
| chr20:30155881:30156027\|30156083:+@chr20:30156923:30157370:+ | HM13 | A5SS | -0.57 |  |  |
| chr18:5238099:5239048:+@chr18:5240186\|5241698:5241811:+ | LINC00667 | A3SS | -0.57 |  |  |
| chrX:133559231-133559360:+@chrX:133559691-133562822:+ | PHF6 | RI | -0.57 | HepG2 | 0.040546 |
| chr19:34712412:34712643:+@chr19:34717313:34717369:+@chr19:34718270:34720420:+ | LSM14A | SE | -0.56 |  |  |
| chr16:15808766:15808938:-@chr16:15802660:15802698:-@chr16:15796992:15797980:- | MYH11 | SE | -0.56 |  |  |
| chr1:110213909:110214004:+@chr1:110214095:110214205:+@chr1:110217369:110217908:+ | GSTM2 | SE | -0.56 |  |  |
| chr1:28907158-28907072:-@chr1:28906493-28906045:- | SNHG12 | RI | -0.55 | k562 | 0.001274 |
| chr9:133993143:133993234\|133993305:+@chr9:133995622:133998539:+ | AIF1L | A5SS | -0.55 |  |  |
| chr17:1617308-1616997:-@chr17:1616189-1614798:- | MIR22HG | RI | -0.55 |  |  |
| chr5:42808253:42808468:-@chr5:42804758:42804875:-@chr5:42799982:42801433:- | SELENOP | SE | -0.55 |  |  |
| chr5:42808253:42808468:-@chr5:42804758:42804875:-@chr5:42799982:42801433:- | SEPP1 | SE | -0.55 |  |  |
| chr8:143746087-143745895:-@chr8:143740356-143738874:- | JRK | RI | -0.55 |  |  |
| chr17:19235166:19235381:+@chr17:19237269\|19238718:19240028:+ | EPN2 | A3SS | -0.55 |  |  |
| chr5:149829319:149829049\|149829266:-@chr5:149827148:149827298:- | RPS14 | A5SS | 0.54 |  |  |
| chr10:72100309:72100458:-@chr10:72083619:72083786:-@chr10:72058729:72061264:- | LRRC20 | SE | -0.54 |  |  |
| chr11:35211382:35211612:+@chr11:35232793:35232996:+@chr11:35236399:35236461:+ | CD44 | SE | 0.53 |  |  |
| chr1:207958416:207958451:+@chr1:207958964:207959027:+@chr1:207966864:207968861:+ | CD46 | SE | -0.53 |  |  |
| chr6:75797293:75797463:-@chr6:75795081\|75796273:75794042:- | COL12A1 | A3SS | -0.53 |  |  |
| chr8:146078452:146078224\|146078378:-@chr8:146075551:146076780:- | COMMD5 | A5SS | 0.52 |  |  |
| chr15:37100525-37100690:+@chr15:37101105-37102449:+ | C15orf41 | RI | -0.52 |  |  |
| chr11:74555213:74555328:-@chr11:74554899:74555004:-@chr11:74551955:74554498:- | XRRA1 | SE | 0.52 |  |  |
| chr15:35280317:35280497:-@chr15:35279758:35279874:-@chr15:35270542:35275691:- | ZNF770 | SE | -0.52 |  |  |
| chr19:7535012-7535201:+@chr19:7535423-7537363:+ | ARHGEF18 | RI | -0.52 |  |  |
| chr17:74722912:74723295:+@chr17:74725772:74725876:+@chr17:74729060:74729297:+ | METTL23 | SE | 0.51 |  |  |
| chr9:124544610-124544680:+@chr9:124545815-124547809:+ | DAB2IP | RI | -0.51 |  |  |
| chr15:75195127-75194945:-@chr15:75194404-75192333:- | FAM219B | RI | -0.50 |  |  |
| chr10:24833910-24834032:+@chr10:24834756-24836772:+ | KIAA1217 | RI | -0.50 | HepG2 | 0.013752 |
